# Biomarkers evaluation in olfactory neuroepithelial cells isolated from Mexican patients with and without Mild Cognitive Impairment

**DOI:** 10.1002/alz.092648

**Published:** 2025-01-09

**Authors:** Maria‐del‐Carmen Silva‐Lucero, Laura Gomez‐Virgilio, Andres‐Ivan Gutierrez‐Malacara, Obed‐Ricardo Lora‐Marin, Maria‐del‐Carmen Cardenas‐Aguayo

**Affiliations:** ^1^ UNAM, School of Medicine, Department of Physiology, CDMX, DF Mexico

## Abstract

**Background:**

Mild cognitive impairment may increase the risk of Alzheimer's disease (AD) or probably accelerate the progression. AD is the most common cause of dementia, substantial neuronal loss, and neuropathological lesions can damage many brain regions. Symptoms of the disease begin with mild memory difficulties and evolve towards cognitive impairment. Novel biomarkers include PET scans and plasma assays for amyloid β and phosphorylated tau, which show great promise for clinical and research use, but may be expensive approaches, here we propose a more affordable approach to detect biomarkers in cells isolated from the olfactory neuroepithelium.

**Method:**

Older adults with signs of Mild Cognitive Impairment and healthy individuals of the same age were recruited. We performed a MoCA Test, a medical interview, and an olfactory test for each volunteer. A noninvasive nasal exudate was performed to obtain, isolate, and characterize Olfactory Epithelial Precursor Cells. Once the cultures were established, different biomarkers were evaluated using the Western Blot technique. We selected the biomarkers studied through a bioinformatic DEG’s analysis previously carried out by our research group.

**Result:**

A cohort of 3 patients with cognitive impairment and 3 healthy controls were recruited. We obtained from all volunteers their clinical history, a MOCA‐cognitive test, blood samples, and the culture of the Olfactory Epithelial Precursor Cells of each patient was established. The following markers were analyzed by Western Blot Amyloid precursor protein, total tau (T‐tau) and phospho‐tau217 (p‐tau217), as well as the AD biomarkers selected by our bioinformatics analysis: calcineurin subunit, a serine/threonine phosphatase under the control of Ca2+/calmodulin; β‐synuclein, which is associated with synaptic degeneration; a‐synuclein; and FKBP prolyl isomerase 1B (FKBP1B), a chaperone involved in age‐related Ca2+ dysregulation in the brain. Our data showed no correlation between p/tau217 and cognitive impairment.

**Conclusion:**

The search for altered genes in mild cognitive impaired patient‐derived peripheral cells will allow us to identify potential early biomarkers of AD that could be validated in blood samples from patients with AD, as a possible affordable diagnostic tool.

